# DOES GENDER INFLUENCE RISK FOR ORTHOSTATIC HYPOTENSION IN OLDER ADULTS WITH SARCOPENIA?

**DOI:** 10.1093/geroni/igz038.2519

**Published:** 2019-11-08

**Authors:** Melissa J Benton, Amy L Silva-Smith, Jefferson M Spicher

**Affiliations:** University of Colorado Colorado Springs, Colorado Springs, Colorado, United States

## Abstract

Older adults with sarcopenia may be at risk for unstable postural blood pressure due to diminished lean mass that plays a role in maintaining fluid volume. Males have greater lean mass, so risk may be mediated by gender. We compared postural blood pressure changes in older men (77.1 ± 2.0 years; n = 15) and women (79.6 ± 2.0 years; n = 13) with sarcopenia before and after an overnight fast. Sarcopenia was defined using the Lean Mass Index (males ≤ 19.0 kg/m2; females ≤ 15.0 kg/m2). Body composition was measured using multi-frequency bioelectrical impedance, and blood pressure was measured lying, sitting, and standing. On Day 1 (normally hydrated) there were significant drops in systolic blood pressure, with an overall decrease of -9.1 ± 2.2 mmHg (p < 0.001) between lying and standing. On Day 2 (overnight fast), postural changes were more profound, with an overall decrease of -14.1 ± 2.8 mmHg (p < 0.001). However, when compared by gender, postural changes between lying and standing remained significant but did not differ between men and women (Day 1: men -8.9 ± 2.5 vs. women -9.3 ± 2.5 mmHg; Day 2: men -14.6 ± 4.6 vs. women -13.6 ± 3.1 mmHg). On both days diastolic blood pressure remained stable. In this group of older adults, significant decreases in postural systolic blood pressure were observed in the early morning fasted condition, increasing the risk for orthostatic hypotension (drop in systolic blood pressure -20.0 mmHg). Interestingly, gender did not influence risk.

